# Aggressive Types of Malignant Thyroid Neoplasms

**DOI:** 10.3390/jcm13206119

**Published:** 2024-10-14

**Authors:** Maria Boudina, Eleana Zisimopoulou, Persefoni Xirou, Alexandra Chrisoulidou

**Affiliations:** 1 Department of Endocrinology, Theagenio Cancer Hospital, 54639 Thessaloniki, Greece; mariaboudina@gmail.com (M.B.); el_zisimopoulou@yahoo.gr (E.Z.); 2 Department of Pathology, Genekor S.A., 15344 Gerakas, Greece; pxirou@gmail.com

**Keywords:** differentiated 1, poorly differentiated 2, differentiated high-grade 3, anaplastic thyroid cancer 4

## Abstract

Differentiated thyroid cancer (DTC) includes many subtypes, which demonstrate favorable to aggressive behavior. During the past decades, efforts have been made to describe aggressive thyroid cancers. Within DTC, aggressive variants constitute rare entities with unique histopathological features and compromised survival, as local and distant metastatic disease is frequent. In recent years, the distinct category of poorly differentiated thyroid cancer was introduced in 2004 and the type of differentiated high-grade thyroid carcinoma was recently added in the 2022 WHO classification of thyroid neoplasms. Finally, anaplastic thyroid cancer exhibits a rapid, resistant to therapy, progression and confers the shortest survival. In this review, we will present the characteristics of these thyroid cancer types and also discuss the treatment, management, and follow-up of these difficult cases. Emphasis was given to recent bibliography of the last decade.

## 1. Introduction

Thyroid cancer arising from follicular epithelial cells represents the commonest endocrine malignancy. Most of these neoplasms are well differentiated and have a favorable prognosis. Under the term differentiated thyroid cancer (DTC), the commonest subtypes of papillary (PTC) and follicular thyroid cancer (FTC) are included, representing more than 90% of thyroid cancer. However, within DTC cases, tumors of poor differentiation have long been recognized, differing in terms of histology, clinical behavior, metastatic potential and prognosis from the classic DTC tumors [1]. These aggressive forms have been associated with local infiltrative disease, local recurrences, and distant metastases [2]. Although histological uncertainty may exist in some cases, the identification of these thyroid cancer types is important for the clinician, as patients with aggressive forms of TC require close follow-up and meticulous management.

In recent years, poorly differentiated thyroid carcinoma (PDTC) and differentiated high-grade thyroid carcinoma (DHGTC), represent two, recently reclassified, distinct entities with overlapping clinical phenotypes. Their incidence seems to be less than 5% of all thyroid malignancies [3]. PDTC and DHGTC constitute two separate, nevertheless similar entities, placed under the term high-grade follicular-derived thyroid carcinoma (HGFDTC) [4,5].

**Figure 1:**
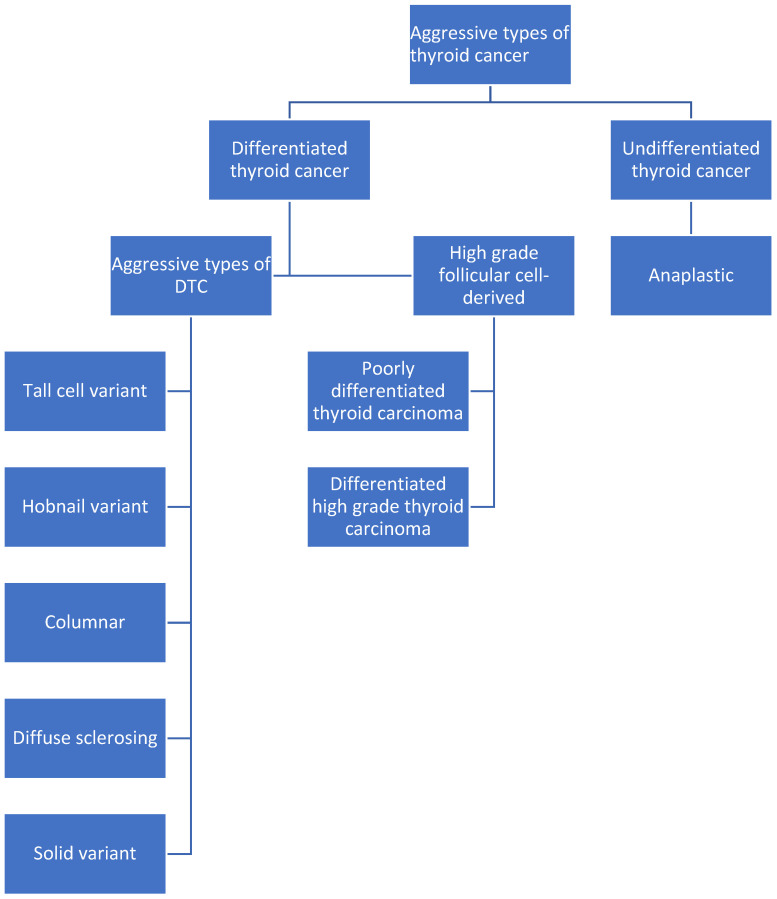
Classification of aggressive types of thyroid cancer.

In summary, aggressive types of thyroid cancer can be categorized into three different entities (Figure 1):

1. Aggressive subtypes of DTC (tall cell, hobnail, diffuse sclerosing, solid and columnar)
2. HGFDTC consisting of (a) PDTC and (b) DHGTC
3. Anaplastic thyroid carcinoma

## 2. Aggressive Subtypes of DTC

### 2.1. Tall Cell Variant of PTC

Tall cell variant (TCPTC) was first described in 1976 [6]. TCPTC represents 5–11% of all PTC cases [7] and is usually seen in older patients. Female patients are also 2- to 3-fold more likely to develop TCPTC [8].

Histologically, TCPTC is characterized by the presence of tall cells with eosinophilic cytoplasm and nuclear features of classic PTC (nuclear grooves, pseudo-inclusions, and overlapping) [1,7]. Tall cell is a rectangular cell, whose height is three times its width. For the safe characterization, 30% or more of tumor cells with a specific morphology are required within the tumor. The proportion of tall cells within the tumor has been a matter of debate and has changed from the 2004 classification (where 50% of tall cells were required) to the 2007 classification (where 30% were considered necessary to establish the diagnosis). Recently, the 2022 classification recommends that pathologists report the presence of tall cells, even in small proportions, as they may define an aggressive PTC [2].

TCPTC usually presents as a large tumor, often multifocal and bilateral, with extrathyroidal extension and advanced TNM stage [7]. These tumors are metastasized in lymph nodes and distant sites and compromise survival, with a 10-year mortality of around 22%. Due to local invasiveness, higher rates of postsurgical positive margins are seen [9]. In many studies, TCPTC appears as an independent risk factor affecting recurrence and survival [10]. However, in other studies, after adjusting for stage and grade, the presence of TCPTC did not predict the recurrence of disease or survival in some studies [11]. The clinical behavior and prognosis of these tumors probably depend on histological aggressive features rather than the presence or the proportion of tall cells, as a large study of more than 3000 tumors indicated [12]. In this study, patients were divided into three groups: PTC with less than 30% tall cells, with 30–49%, and with more than 50% tall cells. Gross extrathyroidal extension, multifocality, pathological lymph nodes and vascular invasion were high in all groups, and remarkably the group with low tall cells had similar indices with the group with more than 50% tall cells [12]. Despite ongoing research, a question that remains to be answered is whether TCPTC without or with aggressive histological features behave differently. In that respect, tall cells in histology may “upgrade” a PTC without aggressive characteristics that was otherwise considered low risk [13]

Although studies are inconsistent regarding mutational status, *BRAFV600E* has a high prevalence in most reports [6]. In one recent study, 93% of TCPTC (28 out of 30 patients) were positive for *BRAFV600E* [14]. As a consequence, RAI refractory disease can be explained in those cases of TCPTC. *TERT* promoter mutations are present in TCPTC in about 7% [15]. These mutations are rare and may co-exist with BRAF mutations. Relapse-free survival is shortened in *TERT* mutated cases versus wild type (16.13 ± 6.15 months vs. 101.34 ± 9.09 months), as well as overall survival and tumor specific survival [12,15].

### 2.2. Hobnail Variant of PTC

Hobnail PTC (HPTC) is a rare variant, with a prevalence of 0.3–2.7% of PTC in different reports. A hobnail PTC consists of at least 30% of hobnail cells, organized in a micro-papillae pattern. These cells have a characteristic appearance with loss of cell polarity and apically placed nucleus, producing a surface bulge that allows the hobnail to form in these cells [16]. An eosinophilic cytoplasm and a low nucleus/cytoplasm ratio are also seen [17].

The first report on a moderately differentiated thyroid cancer with hobnail features was published in 2010, describing a group of eight patients with a rare aggressive form of PTC [18]. Since then, case reports and small series on HPTC have been published. HPTC was included for the first time in the 4th Edition of the WHO Classification of Tumours of Endocrine Organs, where it was considered an aggressive tumor with an epithelial–mesenchymal transition [19]. A year later, the Italian consensus guidelines suggested reporting the presence of hobnail cells in histological thyroid specimens, defining the importance of these cells, even in small amounts, in producing an aggressive tumor [20]. In the 2022 classification, HPTC is considered a thyroid tumor with a poor outcome [4].

*BRAFV600E* is the commonest mutation in HPTC, but many other genetic alterations have been reported with geographical differences [20]. Mutations present in HPTC are in *TP53*, *TERT* promoter, *PIK3CA*, *CTNNB1*, *EGFR*, *AKT1*, and *NOTCH1* genes [21,22]. Multiple mutations within each of these tumors may lead to a more aggressive behavior; therefore, the identification of a specific genetic signature would prove helpful for more efficient management and a better prognosis.

HPTC exhibits an aggressive clinical behavior, with local recurrences in 10–83% and distant metastases in 25–100% of cases in major HPTC studies [23]. Clinically it may present in various ways, from an incidental finding to a locally advanced tumor with compressive symptoms [16]. These tumors are often difficult to treat, as only one third of HPTC patients have RAI avid tumors [21].

### 2.3. Solid Variant of PTC

Solid variant of PTC (SVPTC) represents 1–3% of all PTC. In 1985, Carcangiu et al. found that PTC tumors with solid components had more often lymph node and distant metastases without compromising survival [24]. Some years later, the high prevalence of SVPTC in children with PTC after the Chernobyl nuclear accident was found [25], as well as the relation of SVPTC and radiation pediatric PTC with *RET/PTC3* re-arrangements. Nevertheless, during the following years, it was noted that the presence of SVPTC was not strictly exclusive to radiation exposure.

SVPTC consists of solid, trabecular or insular elements in hypercellular nests within the tumor, with absent or <50% presence of papillae [26]. However, cytological features of PTC are retained. The proportion of solid cells in histological material is important for the diagnosis of SVPTC, as 50–70% of solid cells were considered necessary by researchers [24,27]. Although this issue has not been clarified, and because PTC may have solid elements, solid architecture in >50% of the tumor is probably essential for correct diagnosis [28]. Additionally, in the 4th Edition of the WHO Classification, SVPTC was considered a tumor with solid growth without increased mitoses and absence of necrosis [29]. The differential diagnosis of SVPTC involves poorly differentiated thyroid carcinoma, follicular variant of PTC, and follicular carcinoma with a solid pattern [1], and therefore the distinction between these entities is important.

SVPTC expresses a specific genetic profile, with nearly 70% of these tumors possessing *RET/PTC* re-arrangements and less frequently *BRAF* (common and uncommon mutations) and *RAS* mutations [30]. RAS mutations are seen in 54% of encapsulated tumors [3]. *ETV6-NTRK3* fusions may also be seen in over 40% of cases [26].

SVPTC has been associated with conflicting evidence regarding its behavior, from recurrence and metastases similar to PTC [3], to aggressive behavior in local and distant sites [31]. Cancer mortality was higher than classic PTC in the latter meta-analysis looking into 11 relevant studies [31]. As vascular invasion and extrathyroidal disease is present in one-third of patients, these tumors require attention and close follow-up.

### 2.4. Diffuse Sclerosing Variant of PTC

Diffuse sclerosing thyroid cancer (DSTC) was first described in 1985 and accounts for around 6% of PTC [32]. It is often seen in young patients. It demonstrates a diffuse involvement within the gland, often bilateral [33]. DSTC has been associated with Hashimoto’s thyroiditis in 30–75% of cases [34]. Because of the diffuse pattern of infiltration, hypothyroidism becomes apparent before surgery in 10% of patients, but hyperthyroidism may also be present [35].

DSTC typically presents as an enlargement of the thyroid gland, with or without a thyroid lesion. Microcalcifications, hypo- and hyper-echoic areas within the gland and presence of affected lymph nodes at diagnosis, may be present [36].

The tumor is histologically characterized by marked metaplasia, diffuse interstitial fibrosis, and generalized lymphocytic infiltration [33,35]. Psammoma bodies are present and nuclear features of PTC are also seen. The extensive infiltration with lymphocytes makes the differential diagnosis between DSTC and thyroid lymphoma difficult.

DSPTC carries various genetic alterations, with most frequent the RET fusion in 32%, followed by *BRAFV600E* mutations in around 20%, and the novel gene for thyroid cancer, USP8 in 10% [37]. *KRAS* and *TERT* mutations are also seen in DSPTC [32].

DSTC displays a more aggressive course than classical PTC, with invasive tumors, multifocality, extrathyroidal extension, vascular invasion, lymph node involvement, and lung metastases [32]. However, according to the 2015 guidelines for DTC, DSTC is not considered a high-risk PTC, like tall cell; instead, it represents an intermediate-risk tumor in terms of recurrence and metastases [38]. Information from the SEER population showed that DSTC in patients younger than 55 years old has an excellent prognosis, despite the aforementioned aggressive features [39].

### 2.5. Columnar Cell Variant of PTC

Columnar cell variant of PTC (CCPTC) is rarely seen, as only 0.15–0.20% of PTC falls into this category [40]. Since its first description in 1986 by Evans, many but small series have attempted to clarify its behavior [41]. CCPTC presents with great heterogeneity, from small indolent to large rapidly growing thyroid tumors [42]. The typical ultrasonographic appearance is a solid hypo-echoic thyroid nodule. The indolent CCPTC tumors are usually small, confided to the thyroid, and are seen in young patients. However, CCPTC tumors tend to be larger in patients older than 65 years and in males [43]. The encapsulated forms of CCPTC are usually less aggressive than the tumors without capsule, although in general CCPTC holds a poor prognosis [44].

Histologically, the tumor is characterized by papillae with pseudo-stratified columnar cells and an absence of psammoma bodies [42]. *BRAFV600E* mutations are seen in one-third of patients [45], and less frequently the *BRAFVE1* expression and *AGK*: *BRAF* fusion are found [46]. Mutations have also seen in the *TERT* promoter, *RAS*, *ATM*, *NOTCH1*, *APC*, *ESR1* [45].

Significant differences with classic PTC were found in locally invasive characteristics, lymph node, and distant metastases [43]. A large number of patients with CCPTC were studied from the SEER database. In the 986 patients who were identified with this PTC variant, the mean survival was 44.06 ± 33.01 months, significantly lower than for those with classic PTC and FTC [47].

### 2.6. Treatment Outline in Aggressive Types of PTC

The treatment of all aggressive variants of DTC is similar. By ATA class of risk, these tumors have an intermediate risk [38]. Surgery represents the cornerstone of therapy. The extent of surgery depends on the size of the tumor and the presence of lymph node metastases, and these subtypes are difficult to diagnose by FNA pre-operatively. The common surgical treatment is total thyroidectomy with central lymph nodes dissection, and lateral lymph nodes removal is additionally performed when a positive lymph node FNA is found [2]. Local recurrences (22% for TCPTC and up to 36% for HPTC) [2] are also considered for re-surgery during follow-up, in order to eliminate cervical disease and increase efficacy of RAI therapy. Even tumors smaller than 1 cm carry a significant risk of positive central lymph nodes, extrathyroidal extension, and local recurrence [48]. On the other hand, it is important to segregate indolent cases among this group, as for example with encapsulated small CCPTC or small TCPTC without aggressive histological features, and to modify our management accordingly. Treatment of these tumors needs to be decided in a multidisciplinary team, also taking into account the patient’s preferences.

Patients are eligible for RAI treatment, according to international guidelines, and repeated doses may be required. However, as these tumors are often *BRAF* mutated [7,21], RAI-resistant disease may appear [2]. In these instances, local disease can be managed with locoregional therapies (thermoablation, cryoablation, percutaneous ethanol injection, laser ablation). Although differences exist regarding protocols and efficacy, all treatment modalities prove safe and effective in reducing lymph node size [32]. A large size greater than 5 cm limits the efficacy of treatment, and better outcomes appear for sizes around 2 cm [49]. Radiotherapy is used in locally invasive tumors not amenable to surgery, and in brain and bone metastases [50].

Distant and cervical metastatic disease that deteriorates and exerts local infiltration in vital structures is a candidate for tyrosine kinase inhibitors treatment (TKI’s). Sorafenib and lenvatinib are FDA approved for DTC patients. In addition, *BRAF* positive tumors can be treated with a combination of dabrafenib and trametinib. The follow up during TKI therapy is described in more detail in the treatment of high-grade tumors.

## 3. High-Grade Follicular Cell Derived Thyroid Carcinoma (HGFDTC)

### 3.1. Poorly Differentiated Thyroid Carcinoma

Poorly differentiated thyroid carcinoma (PDTC) represents a rare but aggressive thyroid malignancy, originating from follicular epithelial cells [51]. It is placed biologically between well-differentiated thyroid cancer, and the highly aggressive undifferentiated anaplastic thyroid carcinoma. Sakamoto et al. were the first to propose PDTC as a separate entity in 1984, and one year later it was named ‘insular’ based on the histological features described, the characteristic well-defined nests (insulae) [52,53]. Twenty years later, in 2004, PDTC was officially established as a separate pathology subcategory and was included in the classification of thyroid tumors by the WHO [53]. In 2006, in Turin, a renewed definition of PDTC was given by pathologists, emphasizing on the growth pattern of the lesion (trabecular, insular, solid). More specifically, according to the Turin criteria, PDTC is characterized by the presence of invasion of the malignant neoplasm (although subset may lack invasion); solid-trabecular-insular growth (well defined, elongated nests surrounded by thin fibrovascular septa); absence of conventional nuclear features of papillary thyroid carcinoma; along with at least one of the following three criteria: convoluted nuclei, tumor necrosis, and mitosis of ≥3/2 mm^2^. Importantly, the nuclei are often raisinoid and hyperchromatic; cytoplasm is usually scant but can be oncocytic; intranuclear cytoplasmic inclusions are absent [54,55]. It was not until 2017 that the WHO classification of Tumors of Endocrine Organs adopted the Turin criteria for PDTC and indicated that any poorly differentiated component in histology should be mentioned in the pathology report [56].

The incidence of PDTC is not well established due to sparse clinical data and differences in histopathological interpretation, but it is estimated to account for 3–5% of all thyroid carcinomas [57,58,59]. There has been noted a geographic variation as reported by Sanders et al. [60], where the incidence was 2–3% in North America, but up to 15% in Northern Italy [61]. The mean age of diagnosis is 55 to 65 years and there has been reported a slight female predominance [56].

The diagnosis of PDTC, as implied above, has been challenging over the years because of this transitional nature between well- and un-differentiated thyroid carcinomas. From a clinical perspective, a rapid cervical growth, due to enlargement of the thyroid and lymph nodes, is the most common sign along with hoarse voice, dyspnea or dysphagia if the laryngeal nerve, trachea, or esophagus is invaded. The patient may have a history of a longstanding uninodular or multinodular thyroid. Since these are also symptoms of other aggressive forms of thyroid cancer, and, since the diagnosis of PDTC is rarely made by fine needle aspiration (FNA), preoperative diagnosis is often delayed. In the majority of cases, 60–70%, the cancer extends to the perithyroidal soft tissues and there is vascular invasion in up to 90%. Furthermore, 85% of the patients who succumb to the disease already have distant metastasis, implying the aggressive biological behavior of PDTC [62]. Additionally, distant metastatic foci are estimated to occur in 40–70% [63]. The most common site of distant metastases is the bone and lungs [64].

PDTC accounts for a small portion of thyroid carcinomas but contributes to a significant proportion of thyroid carcinoma-associated deaths. However, due to sparse clinical data the clinical outcomes remain unclear. A recent meta-analysis, conducted by Kim et al. determined the five-year disease-free survival (DFS) and the overall survival (OS) in 1914 patients diagnosed with PDTC between 2007 and 2023. Overall, five-year DFS and five-year OS were 49.4% and 73.8%, respectively. Meta-regression analysis was further conducted to evaluate the prognostic factors, according to which extrathyroidal extension (ETE) was a major negative prognostic factor for OS [65]. Other prognostic factors that affect OS include tumor size, patient age, and undoubtedly, the presence of distant metastases.

From the perspective of genomics, it is difficult to study the molecular basis because PDTC shares common characteristics with other thyroid cancer subtypes (FTC, ATC). However, there have been a few studies that examined the molecular alterations that take place in PDTC. The most frequently encountered mutations described, are these of *RAS* genes (*H/N/KRAS*), oncogenes related to cell proliferation and survival, which are part of the Ras/Raf/MAPK pathway. In addition to the *RAS* gene, the *TERT* promoter gene is frequently encountered (up to 40%), which is related to an even more aggressive behavior, presenting with distant metastases and leading to higher mortality rates [57,66]. In a recent study conducted by Lee [67], 15 patients diagnosed with PDTC had their genomic profile analyzed. *RAS* gene mutation was most frequently seen and no *BRAF* mutation was found in these patients. However, other studies report *BRAF* mutations up to 28% and *p53* mutations up to 25% [62]. There is a significant relation between mutations and clinical characteristics of the tumor [55]. Interestingly, PDTC presents a high grade of recurrence and metastasis compared to FTC or PTC [68], especially if there is vascular invasion which has been reported more common in *ABCA12*, *ATP13A3* and *CLIP1* somatic mutations [67].

### 3.2. Differentiated High Grade Thyroid Carcinoma (DHGTC)

DHGTCs are tumors with certain characteristics, which demonstrate aggressive behavior similar to PDTC and do not fulfill either the Turin Criteria or the criteria for anaplastic thyroid carcinoma (ATC) [69,70]. Their architectural and nuclear features exhibit increased mitotic activity and/or necrosis. They should be suspected in case of gross, widely infiltrative tumors with solid, fleshy, hemorrhagic or necrotic areas. In some cases, solid satellite nodules invade the surrounding thyroid parenchyma. Moreover, they may have retained PTC-related nuclear atypia or a follicular growth pattern and characteristics not indicative of PDTC [55,71].

DHGTCs are defined as thyroid follicular cell derived malignancies with greater than or equal to 5 mitotic figures per 2 mm^2^ and/or necrosis. They may retain their distinctive architectural or cytological features related to differentiated thyroid carcinomas (papillary, follicular, or oncocytic carcinomas [4,5,72]. It is believed that aggressive subtypes of DTC, widely invasive FTCs, and widely invasive OTCs, progress to DHGTCs, so high-grade areas are a common finding in such tumors. In such cases, it is crucial for the diagnosis to quantify the relative proportion of high-grade morphology in order to choose the proper treatment modalities. A detailed pathology report should refer to the high-grade characteristics, the grade of differentiation, and especially the least differentiated tumor component even if non-predominant [51,73,74].

According to a meta-analysis by Poma et al., DHGTC is often encountered in FTC and in some aggressive subtypes of PTC, mainly solid/trabecular, tall cell, and hobnail, which frequently exhibit extrathyroidal extension, lymph node involvement, distant metastases, and increased risk of recurrence [75]. It is worth noting that these clinical behaviors present even if high-grade features constitute a limited proportion of the neoplastic tissue [2,76]. Moreover, progression to high-grade differentiated morphology may be exhibited in lymph node metastases or at distant sites [77].

Ki67 index may be of value in identifying aggressive behaviors among histological groups not fulfilling the DHGTC criteria [78]. Thereafter, classic, diffuse sclerosing, and follicular subtypes of PTC are less likely to be reclassified as DHGTC [75]. Totally encapsulated tumors satisfying the diagnostic criteria for NIFTP may rarely lead to differentiated high-grade follicular variant PTC diagnosis [72,79].

DHGTC is not common among children, however it has been described in teenagers with fatal consequences. The majority of patients are over 45 years old, with a slight female prevalence and have a history of multinodular goiter. They present with sudden voice changes and/or difficulties in swallowing or eating disorders, due to infiltration of the tumor to adjacent organs such as the trachea or esophagus. During clinical examination, they usually exhibit advanced local disease, with large invasive tumors, extrathyroidal extension, and distant metastases at the time of diagnosis [38,80].

It has been reported that approximately 20% to 50% of patients develop metastatic disease, including those with metastases at the time of initial diagnosis. Moreover, there are patients with an otherwise very small thyroid malignancy, presenting distant metastases [79,81]. Recent studies have reported 5- and 10-year disease-specific survival rates of 70 and 56%, respectively [54,72,82,83,84,85].

DHGTCs may be *BRAF*- or *RAS*-driven lesions, which demonstrate whether the preceding lesion was a PTC or an FTC [72]. DHGTCs are mostly derived from BRAF-driven PTCs, while PDTCs often show aberrant RAS signaling, proving a relationship between FTCs and FVPTCs. Other genetic alterations presenting in HGDTCs and PDTCs, include *TERT* promoter and *TP53* gene mutations and *NTRK* fusions, especially in young patients [72,86]. They may also express mutations in *DICER1* in young populations, which is in contrast to *DICER1* mutations, presented in older adults that tend to be of low-risk histology and have a slow clinical course [87].

### 3.3. Treatment Outline of HGFDTC

Dealing with patients with HGFDTC is very challenging, due to its aggressive behavior. Considering its rarity, there are no established uniform recommendations regarding the optimal management of this entity in the literature. Staging is crucial for disease prognosis and treatment decisions. The mainstay initial therapeutic approach is total thyroidectomy and lateral neck dissection. In case of tumor invasion into surrounding tissues (trachea, esophagus, larynx) removal of gross extrathyroidal extension should be considered, if possible [85]. Other modalities, such as esophageal or unilateral nerve resection or surgery, might be required in order to prevent life-threatening events, such as an airway obstruction or hemorrhage. It seems rational to utilize ^131^I therapy after surgery, in case of potential uptake from the metastatic lesions, although this approach might be ineffective. The American Thyroid Association encourages that patients with aggressive tumor histology should receive high dose (100–200 mCi) of ^131^I, although both PDTC and DHGTC are considered radioiodine refractory thyroid carcinomas [72,80,85,88]. In cases of non-iodine avid disease, as well as in cases of unresectable or metastatic disease, adjuvant treatment might be necessary [57,89]. Such modalities include (1) EBRT, (2) radiofrequency or laser ablation in cases of trachea infiltration, (3) endotracheal stent in cases of trachea obstruction, (4) bone or lung metastasectomy in cases of minimal metastatic lesions, (5) chemoembolization or radiofrequency or laser ablation of hepatic lesions, (6) ethanol injection in infiltrated cervical lymph nodes, (7) cementoplasty in cases of osteolytic metastases, or combinations of the above [38,54,57,75,84,90]. Sanders et al. [60] suggested that EBRT should be considered in patients with tumors larger than 4 cm, extrathyroidal extension, lymph node metastasis, or incomplete surgery [91]. So et al. [91], studied retrospectively 32 patients with DTC, who received EBRT. They concluded that EBRT may control locoregional disease and forms a good palliative option in such cases. Furthermore, EBRT could be utilized in bony metastatic disease [92]. However, in cases of metastases with high risk of local complications, stereotactic radiation or thermal ablation, if feasible, should be an alternative [38,93].

Up to date, systemic medical therapy should be reserved as a last step for the treatment of refractory thyroid cancer patients. Surgery, thyroxine suppression, radioiodine treatment, and local therapies should precede it. It should be offered to symptomatic patients, in life threatening events or in clinically rapid deterioration of thyroid cancer [94,95,96]. Local therapies could also be administered during systemic therapy, by holding the latter for a few days, if necessary. 

During the last decade there has been an evolution in the treatment of resistant/refractory thyroid cancer, by inducing targeted therapies (anti-VEGF, anti-RET, anti-PDGF etc.), the so-called Multiple Kinase Inhibitors (MKI’s). They are cytostatic drugs that cause cessation of cell cycles in different sites of intracellular pathways, by inhibiting angiogenesis. Sorafenib and lenvatinib, have been approved for the treatment of metastatic, RAI refractory, radiologically progressed, differentiated thyroid cancer. Lenvatinib, is superior to sorafenib in progression free survival (pfs) compared to placebo (18.3 vs. 10.8 months) [96,97]. As a second-line treatment in disease progression under VEGFR-targeted therapy (lenvatinib or sorafenib), cabozantinib is considered effective, according to COSMIC-311 Trial [97].

Patients receiving these treatments should monitor their cardiac function, clinical condition, and biochemical and radiological status closely. The most common side effects of lenvatinib are: arterial hypertension, gastrointestinal disturbances, generalized constipation, arthralgias, hypothyroidism, and, less frequently, albuminuria and QT prolongation. Respectively, the most common adverse events of sorafenib include hand and foot syndrome, hypothyroidism, and mucositis. Depending on the severity of the complication (grade), a dose reduction or temporary interruption of treatment may be necessary. Upon clinical improvement, it is usually resumed at a reduced dose.

“Escape” from therapy is the main reason for low PFS (10–18 months). When this is documented by clinical, biochemical, and imaging deterioration, the tyrosine kinase inhibitor is replaced or followed by second-line therapy, as mentioned above [97].

Mutations in *BRAFV600 E*, *RET* or *NTRK* rearrangements are encountered in patients with DHGTC, whereas *RAS* and *TERT* gene mutations are met more often in PDTC patients [55,66,67]. The BRAF inhibitor dabrafenib, with or without the MEK inhibitor trametinib, exhibited encouraging overall survival (OS) and PFS outcomes in metastatic BRAFV600E mutated PTC and should be considered in these patients [98].

According to the LIBRETTO-001 trial, the administration of RET-inhibitor selpercatinib, in 19 patients with RET fusion positive DTC previously treated with at least one systemic therapy, showed an overall response rate (ORR) of 79% and a median PFS of 20.1 months [99]. Likewise, the selective RET inhibitor pralsetinib showed an ORR of 84% and a median PFS of 25.4 months in patients previously treated RET fusion-positive DTC [100].

In NTRK fusion-positive DTC, the selective TRK inhibitors larotrectinib and entrectinib have also shown prolonged OS and PFS, with a 24-month PFS of 84% with larotrectinib and a median PFS of 19.9 months with entrectinib [101,102].

In recent years, many clinical studies have been conducted in order to treat iodine-resistant thyroid cancer by administering drugs that in combination, may target different sites of the intracellular pathways [103,104]. Furthermore, in BRAFV600E mutated patients, the MAPK signaling pathway is over activated, leading to a decreased sodium iodide symporter (NIS). In such cases, NIS sensitizing drugs are administered in order to redifferentiate cancer cells to iodine. Finally, immunotherapy in combination with other targeted therapies is studied in clinical trials for thyroid cancer patients [105,106]. Each treatment strategy should be individualized, in accordance with the molecular pattern of each patient, so that ineffective drugs are avoided. After all, this is the challenge of precision medicine [107,108].

In all patients with HGFDTC, serum thyroglobulin may not serve as an accurate marker of disease recurrence, as such cancers secrete less thyroglobulin, due to their cell poor differentiation [85]. Therefore, Fluorodeoxyglucose-positron emission tomography scan (FDG-PET) is recommended by the American Thyroid Association for the initial staging and follow up of such patients [54,72,80,88,94].

### 3.4. Anaplastic Thyroid Cancer (ATC)

ATC is a rare and very aggressive form of thyroid cancer, with historically low survival and cure rates. It originates from follicular cells, with a loss of their differentiation, as well as morphological features of epithelial origin, demonstrated in histological and immunohistochemical examinations [109]. According to WHO classification, ATC is defined as “a highly aggressive malignancy composed of undifferentiated follicular thyroid cells”.

Its incidence is estimated to be one to two cases per million people per year in the USA, though it is reported more often in Europe. ATC tends to affect older people presenting a peak in the sixth and seventh decades of life, with a female to male ratio of 1.5:2 [61,110,111,112,113]. The median survival is approximately 5–6 months, whereas the 1-year survival rate is 10–15% [114,115,116,117]. The vast majority of ATC patients do not survive more than two years after diagnosis, although there are a few reports of 3–10% of patients surviving more than 10 years [118,119]. According to several studies conducted lately, there are certain risk factors leading to ATC, such as low educational level, blood group B, iodine deficiency, obesity, and neglected longstanding goiter [120,121,122,123].

ATCs are classified as stage IV regardless of tumor burden or metastatic profile. They are further subdivided in accordance with locoregional and distant extent. Tumors confined to the thyroid gland are stage IVA, those with extrathyroidal extension and/or infiltration of regional lymph nodes are stage IVB, and lastly the ones with distant metastases outside the thyroid bed are stage IVC [124]. Distant metastatic disease is encountered in about 50% of patients at diagnosis, highlighting its aggressive nature [125]. Common presenting symptoms of ATC include dysphonia, dysphagia, neck or ear pain, dyspnea, and weight loss. Tissue invasion, high mitotic activity and necrosis form some of the most dominant morphological characteristics of ATC [126].

ATC usually presents as a rapidly enlarged solid cervical mass causing dysphonia, dysphagia, neck or ear pain, dyspnea, and weight loss symptoms, due to the invasion of the tumor to adjacent organs. It often demonstrates a long-standing goiter progression, within a few days or weeks in 30% of patients, followed by hard and fixed cervical lymphadenopathy, local invasion and metastases mainly in the lungs, bones, liver or brain. Due to its extremely aggressive course, patients are mostly diagnosed in advanced stages, with either localized (IVa) in 10%, locally advanced (IVb) in 35%, or metastatic (IVc) disease in 55% of patients [106,107,124,127,128]. Clinically, patients may present with dyspnea, dysphagia, and neck pain because of the infiltration of the tumor to the trachea and oesophagus. Superior vena cava syndrome and laryngeal dyspnea is a common finding revealing the gravity of the disease.

The histological features of ATC show a variability in appearance resembling a sarcoma, a squamous carcinoma, or an undifferentiated carcinoma. The tumors consist mainly of spindle, pleomorphic, tumor giant, epithelial/epithelioid cells, though squamoid cells, squamous cells, rhabdoid cells, small cell, osteoclast giant cell-rich, angiomatoid, and paucicellular variants are also found [106,107,124,127,128].

In <5% of ATC osteosarcomatoid, chondrosarcomatoid, or rhabdomyosarcomatoid, are encountered. It is worth noting that in the same tumor there are areas where different histologic characteristics appear. There is a history of differentiated thyroid carcinoma in more than half of patients. The most frequently encountered histological types associated with ATC, are tall cell, classic, and follicular variants of papillary thyroid carcinoma [129,130,131,132,133]. The main histopathological characteristics are, atypical mitoses, tumor necrosis, infiltration of inflammatory cells, mainly macrophages and neutrophils and nuclear pleomorphism [134,135,136,137].

According to the Memorial Sloan Kettering Cancer Center case series (360), in a period of 34 years, the most common histological subtypes were spindle (26%), pleomorphic (23%) and squamous cell (21%). Tumor necrosis was noted in 77%, atypical mitosis in 77%, and neutrophilic infiltration in 71% of the cases. Moreover, the mitotic index was >20 mitoses per 10 high-power fields in 15% of the cases, and Ki67 was not reported. Thyroglobulin and TTF1 were almost always negative (96% and 70%, respectively), whereas cytokeratins AE1/AE3 were found in 67% and PAX 8 in up to 70%, even though the latter is reported even lower (54.4%) in a recent study [138]. PAX 8 could serve as a marker, especially in the squamous type of ATC, for the differential diagnosis of the head and neck squamous cell carcinoma, which is always negative for PAX8 [139].

Recent studies have demonstrated that *TP53* and *TERT* mutations may lead to the tumor progression from DTC to ATC in certain ATC cases with molecular alterations in *BRAF* and *RAS* genes [66,140,141,142]. It is noteworthy that 38% of patients with *RAS* and 75% of *BRAF* mutant ATC, have either a history or a synchronous DTC [61].

According to a recent retrospective multicenter and SEER database study concerning 642 de novo ATCs and 47 ATCs with a DTC component, no statistical differences in survival or clinical course were demonstrated, however, *BRAF* mutation was expressed more often than *RAS* in tumors progressing from DTC [143].

*TERT* promoter and *TP53* mutations are the most frequently encountered molecular alterations in ATCs, followed by *BRAF* and *RAS*, whereas *NTRK* and *RET* fusions are detected in 2–3% of ATCs [61,66]. There is a geographical diversity associated with the frequency of *BRAF* mutations in different continents [140,142,144]. ATC is characterized by infiltration of tumor-associated macrophages (TAMs) in 40–70% of the total tumor mass. TAMs play an important role in immunosuppression, treatment resistance and poor disease prognosis. Due to TAMs infiltration, several inhibitory immune checkpoint inhibitors are highly expressed in tumor samples. [145,146,147,148]. According to a preclinical study by Schurch, PDL-1 expression has been identified in 70% of ATC samples [149].

In Table 1, there is an overview of the clinicopathological characteristics of aggressive types of DTC as well as ATC.

### 3.5. Treatment Outline of ATC

Treatment goals in ATC may be therapeutic or palliative in accordance with the stage at diagnosis, the prognosis, the treatment toxicities, and the patient’s wish. It is crucial to follow a multimodal approach, combining radical or debulking surgery if possible, EBRT, chemotherapy, or targeted therapy [150,151]. Given that ATC is a rapidly progressive thyroid malignancy with locoregional advanced disease and distant metastases often at presentation, initiation of the appropriate treatment is crucial [107,152,153,154].

Surgery, when feasible, is crucial as it provides prolonged survival in patients with stage IVa. EBRT is recommended as an adjuvant therapy postoperatively. The dose delivered (45–60 Gy) seems to predict survival and provides an optimal control, as demonstrated in different studies. It may also be delivered preoperatively in order to enable surgery. It is worth noting that intensity-modulated radiation therapy (IMRT) causes fewer toxicities [155,156]. In cases of stage IVB disease, debulking surgery of the primary tumor, followed by EBRT to the thyroid bed with concomitant radiosensitizing chemotherapy, remains the mainstay of treatment. In patients with stage IVB inoperable tumors or stage IVC disease, systemic therapy should be considered [124,150].

Most studies have demonstrated that concomitant chemoradiotherapy, with either doxorubicin or paclitaxel/docetaxel ± platin, administered in patients without targeted molecular mutations, leads to one-year OS rates between 30% and 50% [157,158,159,160].

The combination of dabrafenib + trametinib has been approved by FDA since 2017 for the treatment of BRAFV600E-mutated ATC [161]. The results of the phase II ROAR basket trial, which showed an objective response rate (ORR) of 56%, a median PFS of 6.7 months, and a median OS of 14.5 months [162]. One approach aiming to prolong duration of response in initially inoperable tumors, has been to administer initially BRAF-directed therapy in order to make the tumor operable and then proceed to surgery [125]. In a retrospective study, this treatment strategy, known as the neoadjuvant approach, led to OS of 100% and 83% at 6 and 12 months, respectively [106,163,164]. There are also reports of 2-year OS of 80.3% in a population composed of 63% stage IVC patients [165]. On the other hand, in non-BRAF-mutated ATC, single agent kinase inhibitors show shorter responses, with a median PFS of 2.6 months with lenvatinib and 1.9 months with sorafenib [162,166].

Immune checkpoint inhibitors (anti-PD-1/PD-L1) have been studied in anaplastic thyroid cancer patients. Single-agent immunotherapy has shown limited efficacy, as expected. Hatashima et al. [167], retrospectively studied 13 patients with ATC treated with anti-PD-1 monotherapy (pembrolizumab or nivolumab). ORR was 16% and median PFS 1.9 months [167]. Likewise, in a prospective phase II trial, an antiPD-1 agent, spartalizumab, showed an ORR of 19% and a median PFS of 1.7 months [168]. According to another phase I study, the combination of durvalumab with tremelimumab and stereotactic body radiotherapy (SBRT), the median OS was only 14.5 weeks in patients with metastatic ATC [169]. On the other hand, a combination of immune checkpoint inhibitors with kinase inhibitors showed more encouraging results, owing to a synergistic effect between these two drug classes [170,171,172].

As for BRAFV600E-mutated ATC, a retrospective study of 71 patients that compared dabrafenib/trametinib alone to dabrafenib/trametinib + pembrolizumab added either at baseline or at time of progression, exhibited significant improvement in survival because of the addition of anti-PD1 immunotherapy. The median OS was 17 months with the three regimens, compared to 9 months with BRAF/MEK inhibitors alone (*p* = 0.037). It is noteworthy that PFS was also significantly improved when an anti-PD1 was added to the initial treatment (Median PFS 11 vs. 4 months; *p* = 0.049) [173].

In cases of non-BRAF-mutated ATC, according to a prospective phase II trial of 27 patients, combining lenvatinib with pembrolizumab showed an ORR of 52% and a median OS of 11 months, compared to a median OS of only 3.2 months with lenvatinib monotherapy [170]. The 2024 National Comprehensive Cancer Network (NCCN) guidelines suggested the induction of pembrolizumab as monotherapy, or in combination with lenvatinib, as a potential treatment option in selected patients with ATC, due to the significant improvement in survival they exhibit [171].

Currently there are multiple clinical trials with various combinations of immunotherapy with other regimens in ATC such as, cabozantinib + atezolizumab (NCT04400474) and pembrolizumab + docetaxel (NCT03360890), among others. An ongoing phase II trial (NCT03246958) of ipilimumab + nivolumab, showed that 3/10 enrolled ATC patients exhibited partial responses, two of which lasted more than one year (13 and 26 months) [172,173]. Pembrolizumab is also being studied as an adjuvant treatment in patients with stage IVB disease after intensity-modulated radiation therapy (IMRT) (NCT05059470).

A multimodal approach is associated with better overall survival. Each patient with ATC should be addressed to a multidisciplinary team, so as to be provided with the best individualized treatment and be given information on the treatment plan and its probable complications. Such situations need a shared decision making with the patient and his family, as the clinical course is difficult, with impacts on the quality of life of the whole family [124,155].

## Figures and Tables

**Table 1:** Clinicopathological characteristics of aggressive types of DTC and ATC.

Cancer Type	Frequency	Prominent Cell Type	Main Difference from PTC	Main Genetic Alteration	Metastatic Potential	10-Year Survival
Tall cell PTC	5–11% of PTC	Tall cells with tumor cell height of at least three times the width, eosinophilic cytoplasm and typical nuclear features of PTC	≥30% tall cells	*BRAFV600E* *TERT* promoter and *TP53* mutations	Lymph nodes and distant metastases (lung, bones)	96.3 [37]–99% [129]
Diffuse sclerosing PTC	6% of PTC	Tumor cells with typical nuclear features of PTC, arranged in solid nests and papillary formations with squamous metaplasia	Diffuse involvement of the gland, dense sclerosis, numerous psammoma bodies, extensive lymphatic infiltration, usual extrathyroidal extension	*RET* rearrangements		99.5% [37]
Hobnail PTC	0.3–2.7% of PTC	Hobnail cells with eosinophilic cytoplasm and enlarged nuclei, bulging from the apical surface	≥30% hobnail cells	*BRAFV600E*		71% [30]
Solid PTC	1–3% of PTC	Tumor cells with variable nuclear features of PTC showing solid, trabecular, or nested growth patterns	>50% solid trabecular growth, with lack of necrosis and high mitotic rate	*RET/PTC3* fusions [35]		96% [69]
Columnar PTC	0.15–0.2% of PTC	Columnar cells with pale to eosinophilic cytoplasm, prominent pseudostratification and occasional subnuclear vacuoles	Neoplastic cells lack the typical nuclear features of PTC Frequent immunο-reactivity of neoplastic cells to CDX2	*BRAFV600E*		93.07% [41] (98.9% in ECPTC)
Poorly differentiated PTC		Neoplastic cells lacking nuclear features of PTC, with solid, trabecular or insular growth pattern At least one of the following three features: Mitotic rate ≥ 3/2mm^2^ Tumor necrosis Convoluted nuclei	Absence of nuclear features of PTC Solid, trabecular or insular growth pattern Tumour necrosis and high mitotic rate	*RAS* mutations *TERT* promoter, *PIK3CA* and *TP53* mutations	Advanced local residual disease and distant metastases (lung, bones)	50% [62]
High grade DTC		Neoplastic cells with any nuclear cytology and papillary, follicular or solid growth pattern One of the following two features: Mitotic rate ≥ 5/2 mm^2^ Tumor necrosis	Tumor necrosis and high mitotic rate	*BRAFV600E* mutations *TERT* promoter, *PIK3CA* and *TP53* mutations	Advanced local residual disease and distant metastases (lung, bones)	72% [129], 56% [62]
Anaplastic TC		Undifferentiated cells (spindle, epithelioid, giant cells) Necrosis, elevated mitotic rate Primary squamous carcinoma of thyroid is now considered a morphologic pattern of ATC	Undifferentiated cells	*BRAFV600E* mutations	Advanced local residual disease, adjacent organs infiltration and Distant metastases (lung, bones)	3% [80]

## Data Availability

The original contributions presented in the study are included in the article, further inquiries can be directed to the corresponding author.
